# Pertaining to Insurance

**Published:** 1876-08

**Authors:** 


We are constantly in receipt of letters from our readers asking for information relative to the various life and fire insurance companies in which they are insured. These inquiries have now become so frequent, and the subject of insurance has become so important to all, we have concluded to devote a limited space to the discussion of the various plans and topics of interest to the public, pertaining to the business, and to answering the communications of our readers. We shall, in the execution of this plan, furnish nothing but information of the most reliable character as to both the standing of companies and their system of doing business, hence our patrons can rely on the correctness of our opinions in all cases and under all circumstances.

---

There are as good fire insurance companies out of the National Board of Underwriters as in it. Membership in the National Board is not a guarantee of solvency and strength.

---

We are unable to answer our correspondent, "California," this month, relative to the practices of the Continental Life Insurance Company, of New York. We have sent for the documents, and will advise further in the matter in our next issue.

---

Tontine policies are not desirable for persons of slender means; the best plan on which such a person can insure is the regular life plan. Endowments *as a speculation* are a delusion.

---

Every man with a family should insure his life in a good company for a moderate amount, on the simple life plan, and promptly meet his payments of premiums as they fall due. But due care should be used in the selection of a company, and all "sharp" and questionable concerns avoided. Much information on this point can be gathered from a careful reading of this department of HALL'S JOURNAL each month.

---

For the benefit of our correspondent, "G. W. W." we would state that the fire insurance company to which he refers has a bad reputation, and frequently figures in the courts as defendant. What little capital it has is badly impaired, and its failure is only a matter of time. There are many good and reliable companies doing business in your State which you can patronize without being compelled to deal with such a frail concern.

---

We receive frequent communications from policy holders in life insurance companies asking our advice as to what is best for their interests in cases where they are importuned to exchange their policies for those upon a different plan or in some other company, or when the company in which they have insured has transferred its business and assets to some other company. To all policy holders desiring information, and who send their address to this office, we will give such facts as we may have.

---

The Union Mutual Life Insurance Company, of Maine, the principal office of which is in Boston, Mass., has for many years ranked among the most reliable and well managed life offices in this country. Within the past month, however, considerable speculation has been indulged in in life insurance circles and among the policy holders as to a change in the management of this company, whereby the president, Mr. H. S. Washburne, retires from that position, and Mr. John E. De Witt, President of the United States Life Insurance Company, of New York, becomes his successor. It is claimed by some that the object of this change is the eventual consolidation of the two companies under one name, while, on the other hand, it is asserted that separate organizations will be maintained and business conducted as heretofore. We shall be able to give full particulars in our next issue, and can only now state that we fully believe the interests of all policy holders will be fairly and amply protected, as the new president is a life insurance expert of large experience and undoubted integrity.

---

Our correspondent, "C. W. R.," of Trenton, N. J., having been solicited to change his policy in a well known company, from an Endowment to a Tontine policy, asks our advice. We would suggest that he make no change, especially to the Tontine plan, as that system generally proves very delusive and unsatisfactory, and in some instances approaches very nearly to a gambling operation.

---

The New York fire insurance companies have declared their July dividends as follows: 3½ per cent., Continental, Standard; 4 per cent., Amity; 5 per cent., Atlantic, Relief, Resolute, Knickerbocker, Hope, Arctic, Guardian, Lamar, Lenox, American Exchange, Adriatic, Firemen's Fund, Home, Hamilton, New York and Yonkers, German American, Mercantile, Lorillard, Niagara, Emporium, Commerce, Hoffman, Irving, Hanover, Germania, Republic; 6 per cent., Importers and Traders', Manufacturers and Builders', Firemen's Trust, Howard; 7 per cent., Manhattan, Ridgewood, Empire City, Firemen's; 7½ per cent., Farragut, Star; 8 per cent., United States, American, National, Safeguard; 10 per cent., Clinton, Citizens' New York, Equitable, Phenix, Stuyvesant, Williamsburgh City,

People's Park, Mechanics', Peter Cooper, Long Island, Mechanics and Traders', Tradesmen's, Globe, Commercial, Kings County.

---

"Policy No. 14,844" in the ——— Life Insurance Company, of New York, wants to know how he can procure an itemized account of his company's assets including a description of its mortgage investments. We would refer him to the Insurance Department at Albany, provided the company itself is unwilling to furnish him with the information he desires.

